# A Third Way for Entomophthoralean Fungi to Survive the Winter: Slow Disease Transmission between Individuals of the Hibernating Host

**DOI:** 10.3390/insects4030392

**Published:** 2013-07-23

**Authors:** Jørgen Eilenberg, Lene Thomsen, Annette Bruun Jensen

**Affiliations:** Department of Plant and Environmental Sciences, University of Copenhagen, Thorvaldsensvej 40, DK 1871 Frederiksberg C., Denmark; E-Mails: lene.thomsen@e-posthus.dk (L.T.); abj@life.ku.dk (A.B.J.)

**Keywords:** *Entomophthora*, *Pollenia*, winter survival, spores

## Abstract

In temperate regions, insect pathogenic fungi face the challenge of surviving through the winter. Winter is a time when hosts are immobile, low in number or are present in a stage which is not susceptible to infection. Fungi from Entomophthoromycota have so far been known to survive the winter in two ways: either as (1) thick-walled resting spores released into environment from dead hosts, or as (2) structures inside the dead host (e.g., hyphal bodies). Here we report, from the Danish environment, a third way to survive the winter, namely a slow progression and transmission of *Entomophthora schizophorae* in adult dipteran *Pollenia* hosts that hibernate in clusters in unheated attics, sheltered areas outdoors (under bark *etc.*). Fungus-killed sporulating flies were observed outside very early and very late in the season. By sampling adults at the time of their emergence from hibernation in late winter/early spring we documented that the fungus was naturally prevalent and killed flies after a period of incubation. Experimentally we documented that even at the low temperature of 5 °C, the fungus was able to maintain itself in *Pollenia* cohorts for up to 90 days. From these observations the full winter cycle of this fungus is elucidated. The three types of winter survival are discussed in relation to fungus epidemic development.

## 1. Introduction

The life cycles of fungi from Entomophthoromycota are closely associated with the life cycles of their hosts. Most species from this Phylum are specialists infecting only closely related taxa and in certain cases just a single host species [1,2,3]. Such high specificity means these fungi are strongly dependent on their hosts. An important feature of Entomophthoromycota is asexual conidia (the infective unit) for rapid infection cycles and thick-walled resting spores for survival of the fungus outside the dead host during winter [1,4,5]. Resting spores are important in temperate climates for survival during periods when hosts are not present in the susceptible stage and/or temperatures are much too low for a conidial infection cycle to take place. A second way of winter survival outdoors is as hyphal bodies in dead hosts [6] or in living, hibernating hosts [7].

*Entomophthora muscae* is a species complex known to infect many different adult dipteran hosts [2,8]. The most well-known species, *Entomophthora muscae*, infects the house fly *Musca domestica* [9,10]. In *M. domestica* the fungus can be transmitted by conidial infections year-round [11], with rapid cycles at room temperature (lethal time of less than one week). This host-pathogen system is characterized by environmental conditions (stables), which can be buffered against extremes throughout the year, and therefore there is basically no need for specific winter survival structures. Thus, it is no major surprise that resting spores from this host-pathogen system are not known [9]. *E. muscae* is also known from *Delia radicum*, an anthomyiid fly that lives outdoors and whose larvae feed on cabbage roots. In this host, resting spores instead of conidia are produced late in the summer in order to ensure the winter survival of the fungus [1]. Other members of the *E. muscae* species complex also produce resting spores, for example *Entomophthora syrphii*, which infects hover flies [12]. 

Another member of the *E. muscae* complex, *Entomophthora schizophorae,* is peculiar in that resting spores produced *in vivo* are not known [2,8,13] despite that the fungus infects dipterans living indoors (e.g., it infects *M. domestica* [14,15] and outdoors in temperate conditions [2,16]. For example, in a two-year study of this fungus in carrot flies, *Chamaepsila rosae*, [16] a high prevalence of *E. schizophorae* was documented but solely in the conidial stage. Apparently, resting spores were not produced by infected hosts, even at low temperatures [17], so winter survival structures in this host pathogen system are unknown. *E. schizophorae* is also known to infect cluster flies, *Pollenia rudis* [2,8]. Flies from the genus *Pollenia* can be found as adults throughout the year in temperate regions [18,19,20]. In winter *Pollenia rudis* hibernates as adults in unheated attics and other parts of buildings [20]. In these places temperatures are low during the winter (but not as low as outdoors and can remain at 5 °C while it is freezing outdoor), and here *Pollenia* can be found in high numbers from November to March or April [21]. In late winter and very early spring the hibernating adult flies gather for a brief period around windows before leaving the building to fly outdoors. As a model system, *Pollenia rudis* and *E. schizophorae* is rather unique: the fungus infects the adult stage of the host and is dependent on the presence of such susceptible hosts. The adult stage of the host is present year around but in highly variable environmental conditions (especially temperature) and in very different physiological conditions, from actively flying, food-seeking and egg-laying individuals during the summer to very little or even no activity among hibernating hosts in buildings and cracks in old tree trunks.

The aim of our study, accomplished through observational and experimental studies, was to test how *E. schizophorae* survives the winter in its adult *Pollenia rudis* hosts. In particular, we aimed to determine if transmission between hibernating hosts at low temperatures takes place. Furthermore, we propose a connection between overwintering of the fungus and the life cycle of the host. This connection to the life cycle of the host adds a third, previously undescribed way in which a fungus from Entomophthoromycota is able to survive the winter.

## 2. Experimental Section

*Morphological features:* Primary conidia were collected from infected cadavers by allowing them to be discharged onto glass slides in a humid chamber. The fungus species was determined based on the shape and size of the primary conidia and number of nuclei per conidium (after staining with lactic acid or DAPI, respectively) [3,22]. Scanning electron microscopy of adult sporulating *Pollenia* was made using a method described earlier [23].

*Late and early sporulating* Pollenia *cadavers:* At irregular intervals, we searched for fungus-killed cadavers in Denmark in attics during winter and outside early and late in the season.

*Sampling* Pollenia *in late winter and incubating individually to assess prevalence*: 265 *Pollenia* were sampled at five different localities in Denmark and Southern Sweden in 2001, 2002 and 2013. In all cases sampling took place indoors at unheated places during the short time interval between late winter and early spring, when *Pollenia* have emerged from their hibernation places and cluster around windows soon ready to fly outdoors. Flies were incubated individually in 25 mL medicine cups with 3% water agar and supplied with a sugar and dry yeast paste at room temperature and checked twice a week. Flies from the prevalence studies that died within two weeks without signs of infection, were dissected to look for resting spores.

Sampling Pollenia in autumn and incubating flies (1) individually to assess prevalence, or (2) in cohorts to assess initial prevalence and further transmission at low temperatures: A total of 525 adult Pollenia spp. were sampled on October 25, 2000 from their hibernating site, an unheated attic in an old farm house, at Ørslev, Zealand, Denmark. In October, temperatures in such attics would typically be approx. 10 °C, then later drop to 5 °C. Of these sampled flies, 284 were individually transferred to 25 mL medicine cups with 3% water agar and supplied with a sugar and dry yeast paste and incubated at 10 °C (72 individuals) or at 5 °C (212 individuals) to assess prevalence and lethal time (measured in weeks after ‘latest potential day of natural exposure’). Also, from these sampled flies, four cohorts of approx. 50 flies were incubated in cages (40 × 25 × 25 cm) and supplied with water and food one day to estimate the initial prevalence and further transmission in cohorts at 10 °C (one cohort) and at 5 °C (three cohorts). The cages were inspected weekly and the presence of fungus killed or dead flies (unknown reason) were recorded. Old, dry cadavers were removed after one week.

*Experimental transmission in cohorts at low temperatures*: Three cohorts of 50 uninfected flies each (sampled late autumn and kept individually in a quarantine at room temperature for more than two weeks to ensure no latent infection) were used in a transmission experiment to elucidate the disease transmission in cohorts when time of initial exposure was known. The flies were incubated in cages (see above) together with three freshly sporulating cadavers, one cage at 10 °C and two cages at 5 °C. The cages were monitored as described above.

## 3. Results and Discussion

Our studies provided a full overview of the winter survival strategy of *E. schizophorae* in its hosts, *Pollenia* flies, and revealed a, yet undescribed, strategy with slow disease development and transmission between individuals of hibernating adult hosts at cold temperatures.

**Figure 1:**
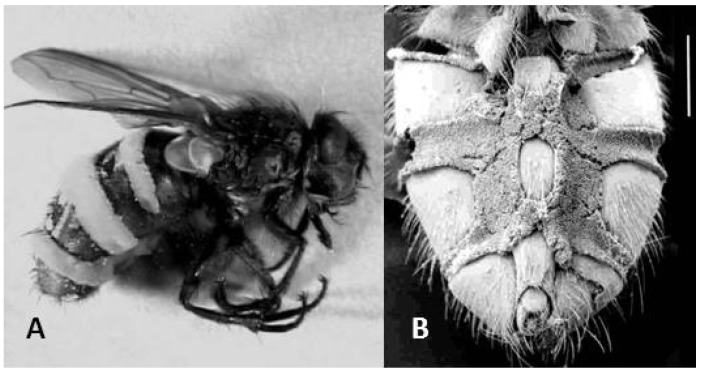
*Pollenia* flies killed by *Entomophthora schizophorae.* (**A**) Fly shown in lateral view, conidiophores visible as almost white intersegmental bands. (**B**) Scanning electron microscopy image from ventral side showing densely packed conidiophores (scale bar 1 mm).

The identity of the fungus killing the *Pollenia* flies was confirmed by morphology of the primary conidia which fell well within the range for *E. schizophorae* [2,8]. In *E. schizophorae* killed flies, the fungus grew out as densely packed, short conidiophores. Sporulation occurred vigorously from both the dorsal (Figure 1A) and ventral surfaces (Figure 1B) of the cadaver. The densely packed conidiophores (Figure 1B) indicate an adaptation of the fungus to infect hosts in close proximity, while dispersal of conidia into open air would potentially benefit from longer conidiophores. 

**Figure 2:**
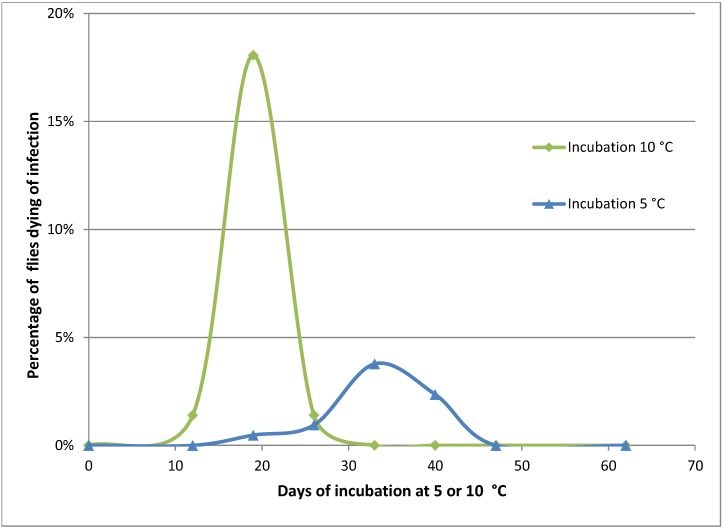
Mortality of *Pollenia* caused by *Entomophthora schizophorae.* Adult flies (284 in total) were sampled October 25, 2001 on a hibernating site (Ørslev, Skælskør, Denmark) and incubated individually at 10 °C or 5 °C.

**Figure 3:**
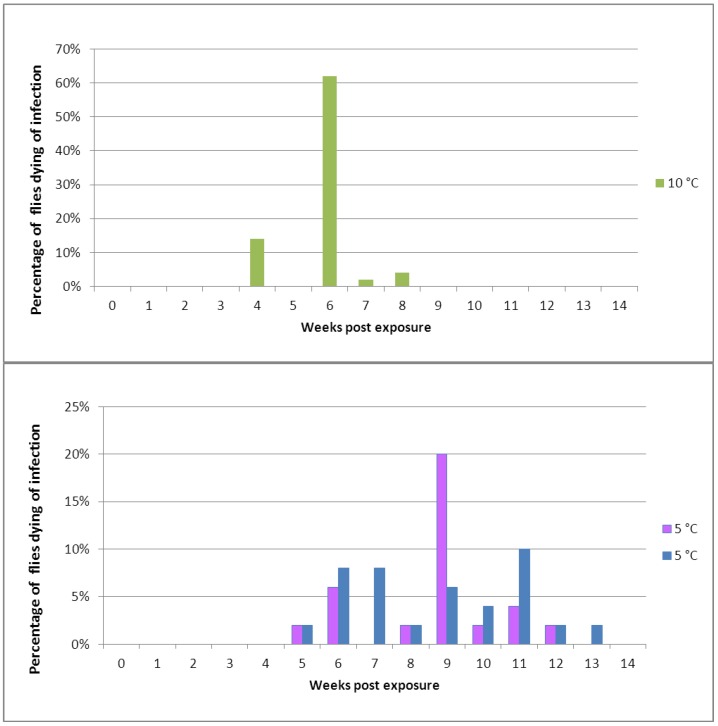
Mortality in cohorts of healthy *Pollenia* exposed to a spore shower of *Entomophthora schizophorae.* In total, 150 Adult flies were subjected to sporulating *Pollenia* cadavers and incubated in cohorts at 10 °C (one cohort) or 5 °C (two cohorts).

In a few cases we found old and desiccated fungus-infected cadavers indoors around window panes during winter at hibernating sites. We were not able to inspect the sites of the clusters without destroying parts of the construction, but the few dry cadavers proved that infectious flies must have been around the overwintering sites. The earliest sporulating *Pollenia* from outdoors was recorded on April 1, 1999, from one specimen collected in Jægerspris, Denmark. At this time of the season the *Pollenia* flies are just about to leave the hibernating sites and the outdoor temperatures (daily average mostly below 5 °C) do not allow an infection cycle to complete itself except after a long period of incubation. Therefore sporulating flies found outdoors very early in the season must have been infected before leaving their place of hibernation. The latest outdoor record of sporulating *Pollenia* was observed November 7, 2009, in one specimen collected in Copenhagen, Denmark. Sporulating flies found so late in the season strongly indicate that upon gathering for hibernation, some adult *Pollenia* are carriers of *E. schizophorae* infection and bring the infection into hibernation places. Thus, sporulating *E. schizophorae*-killed hosts are present outdoors from the very early start of the season immediately after winter and until the very end of the season immediately before winter. This was also confirmed by the incubation of autumn sampled *Pollenia* where both individuals and cohorts proved to be infected by *E. schizophorae* (Figure 2, Figure 3)

Our incubation of flies sampled from indoors during late winter and early spring documented the presence of *E. schizophorae* among hibernating flies (Table 1). In total, we documented 8 cases of infection among the 265 *Pollenia* sampled, revealing an overall prevalence of 3.0% (Table 1); however, there was great variation among the five localities. At Gøderup the prevalence was 4.7% in March 2013 and 25.0% in April 2013, while at Ullared, none of the 89 individuals sampled in February and March 2013 was infected. None out of 83 dissected *Pollenia* contained resting spores.

Naturally infected *Pollenia* sampled in the autumn from one location and incubated individually showed that the disease could develop at low temperatures, 10 °C and 5 °C (Figure 2). When incubated at 10 °C, flies started to die from infections before day 12. A majority of infected flies died before day 19, and after 26 days no flies died from infection. The overall prevalence of *E. schizophorae* in these 72 flies was 20.8%. When incubated at 5 °C flies started to die before day 19, and fungus induced death continued until day 40. The overall prevalence of *E. schizophorae* in these 212 flies was 7.5%. The lethal time at 10 °C may exceed 19 days and at 5 °C the lethal time may exceed 33 days. These lethal times are similar to lethal times for *C. rosae* infected by *E. schizophorae*: at incubation at 11.3 °C, flies died 9–13 days after being subjected to fungus, and at 5 °C, lethal time could exceed 33 days [17]. 

**Table 1:** Prevalence of *Entomophthora schizophorae* in *Pollenia* sampled in late winter and early spring at five hibernacula in Denmark and Sweden and incubated individually at 20 °C.

Locality and sampling date	Number of sampled *Pollenia*	Number of *Pollenia* infected with *E. schizophorae* and % prevalence
Sophienholm, Lyngby, DK, 1/2-2001	13	0
Ørslev, Skælskør, DK, 18/3-2001	24	0
Ørslev, Skælskør, DK, 3/3-2002	9	2 (22.2%)
Granstorp, Småland, SE, 13/2-2013	6	0
Ullared, Halland, SE, 13/2-2013	41	0
Ullared, Halland, SE, 28/3-2013	48	0
Gøderup, Roskilde, DK, 5/3-2013	8	2 (25.0%)
Gøderup, Roskilde, DK, 1/4-5/4-2013	85	4 (4.7%)
Ørslev, Skælskør, DK, 3/4-2013	31	0
Total	265	8 (3.0%)

Our data on *Pollenia* collected in the autumn from one location and incubated in cohorts of 50 flies showed that the disease could be transmitted between flies at low temperatures, 10 °C and 5 °C, if the disease was present (Table 2).

These temperatures can be assumed as representative for unheated attics. The single cage (4) incubated at 10 °C documented an initial prevalence of at least 4.0 %; thereafter, an infection cycle was initiated which resulted in the death of all flies before day 61. In one of the cages incubated at 5 °C, namely cage 2, there was no natural *E. schizophorae* infection, so 47 flies out of 50 were still alive after 110 days. In cages 1 and 3, also incubated a 5 °C, there was an initial prevalence of at least 5.8% and 6.3%, respectively. These naturally infected flies died on approx. day 18 and initiated a transmission at 5 °C, so that an infection cycle at this low temperature was perpetuated. In cage 3, all flies died before day 68, mostly from infection, and in cage 1 the infection cycle continued until day 90.

**Table 2:** Transmission of *Entomophthora schizophorae* in *Pollenia* sampled October 25, 2001 at a hibernating site (Ørslev, Skælskør, Denmark), and incubated in cohorts of 50 flies at 10 °C (cage 4) or 5 °C (cage 1, 2 and 3). In cage 1, 3 and 4 there was an initial natural prevalence of the fungus in the sampled flies, which led to infection cycles. These cycles led ultimately to the complete mortality of the fly cohorts in these cages. In cage 2 there was no initial infection among the flies in the incubated cohort, which survived until the end of the experiment on February 13, 2001.

Date	Days after incubation	5 °C Cage 1		5 °C Cage 2		5 °C Cage 3		10 °C Cage 4	
		Dead without fungus	Dead with fungus	Dead without fungus	Dead with fungus	Dead without fungus	Dead with fungus	Dead without fungus	Dead with fungus
26/10-2000	0	0	0	0	0	0	0	0	0
13/11-2000	18	3	0	0	0	3	0	2	0
20/11-2000	25	0	0	0	0	0	0	3	0
27/11-2000	32	0	0	0	0	1	2	3	0
4/12-2000	39	4	0	0	1	11	3	30	0
11/12-2000	46	13	1	0	0	6	1	1	1
19/12-2000	54	0	0	0	0	9	2	1	0
26/12-2001	61	5	4	0	2	7	0	7	1
2/1-2001	68	11	3	0	0	3	0	-	-
10/1-2001	76	2	1	0	0	-	-	-	-
17/1-2001	83	1	0	0	0	-	-	-	-
24/1-2001	90	2	0	0	0	-	-	-	-
13/2-2001	110	0	1	0	0	-	-	-	-

When experimentally exposed to *E. schizophorae* and incubated at 10 °C, flies started to die 28 days after exposure (Figure 3). Flies experimentally exposed to *E. schizophorae* and incubated at 5 °C started to die 35 days after exposure (Figure 3). Both at 10 °C and at 5 °C the disease transmission continued in the cohort and resulted in an ongoing infection cycle which lasted for 56 days at 10 °C and for 91 days at 5 °C, in concordance with data shown in Table 2.

**Figure 4:**
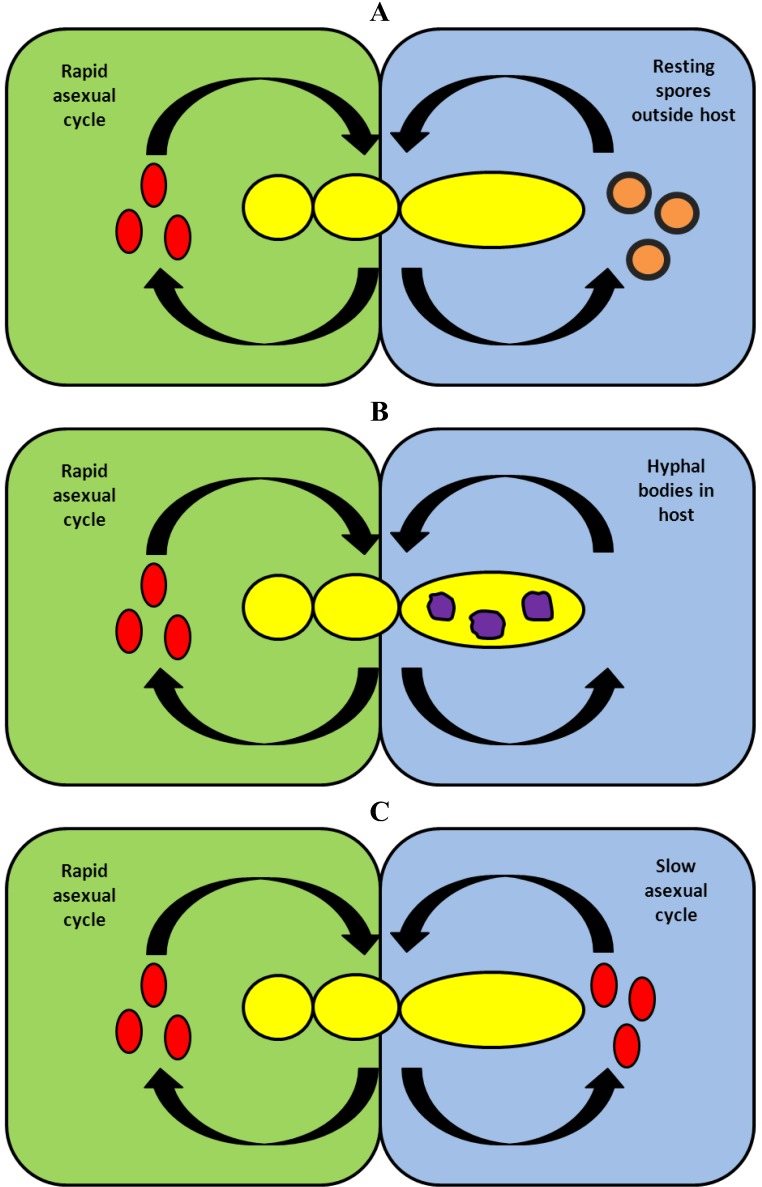
Three strategies for fungi from Entomophthoromycota to survive the winter in temperate climates. In all cases transmission of the fungus disease during spring, summer and autumn, is based on a rapid asexual cycle with conidia. (**A**) During winter, the fungus survives as thick walled resting spores, which after their release are found in the environment outside the host. After winter these resting spores germinate and produce infective conidia. (**B**) During winter the fungus survives outdoors as hyphal bodies inside either a dead host or a living, hibernating host. In the latter case the fungus is present as a latent infection. After winter these hyphal bodies produce infective conidia. (**C**) During winter the fungus survives in cool places indoors by a delayed transmission of conidial infections between individual hibernating hosts. After winter a proportion of these hibernating hosts infected during winter will die and produce infective conidia.

We have illustrated three life history strategies of fungi from Entomophthoromycota in Figure 4. In all cases, the infection cycle during spring, summer and autumn is due to rapidly progressing infections by conidia. During winter, the dominant strategy for the fungus to survive outside the host is to overwinter as thick walled resting spores (Figure 4A). This is known for many host-pathogen systems involving fungi from Entomophthoromycota: such as *Entomophaga maimaiga—Lymantria dispar* [4,24], *Entomophthora muscae—Delia radicum* [1,25], *Strongwellsea castrans—Delia radicum* [1] and others [8]. A major advantage of this method of winter survival is that resting spores are resistant to abiotic factors such as subzero temperatures and drought; this resistance allows them to persist for months. There are two additional advantages to resting spores: First, they can sometimes be dispersed into soil where many host insects emerge as adults in the spring, and second, because they are the product of sexual reproduction, they maintain genetic variability. It is, however, questionable whether sexual reproduction takes place in all species [26]. A disadvantage is that resting spores may need a dormancy period [27] in order to germinate and to produce the infective units. Also, the presence of suitable hosts may trigger the germination of resting spores in spring [28]. A second method of winter survival is shown in Figure 4B. Here, the fungus is present in living or dead hosts as hyphal bodies or similar structures. When temperatures rise, these fungal structures can germinate and produce conidia. This winter survival strategy is known from *E. planchoniana* and its host *Drepanosiphum acerinum*, [6], where hyphal bodies were found in dead cadavers. It is also known from *Neozygites floridana* in the host *Tetranychus urticae* [7]. Here, the fungus survives outdoors in living, hibernating mite hosts, but the study did not include observations if transmission occurred between hosts during the winter. An advantage of this strategy is that once there is a temperature increase, hyphal bodies can quickly germinate and be transmitted to uninfected hosts. A disadvantage is that hyphal bodies are sensitive to external factors outdoors during a long winter.

The *Pollenia—E. schizophorae* host-pathogen system has revealed a third way for fungi from Entomophthoromycota to survive the winter (Figure 4C). The fungus survives the winter by a slow disease development and a slow transmission of the disease among hibernating hosts at temperatures which are low, but not as extreme as outdoor temperatures. Such winter survival can only work when the susceptible stage of the host is in the hibernating stage. This is a relatively rare strategy with at least two advantages: transmission during winter ensures survival of the fungus and, the prevalence of the disease among flies leaving the hibernation sites increases the chance of an early initiation of an infection cycle during spring and summer. There are two possible disadvantages to this strategy: the risk of local extinction of the fungus, as seen from the flies which were sampled and incubated, and the apparent lack of resting spores and sexual reproduction since the fungus apparently relies entirely on conidial, asexual reproduction. It cannot be excluded that genetic recombination can occur by parasexuality or that resting spores occur under certain circumstances. In a study [29] the induction of resting spore formation *in vitro* of *E. schizophorae* (isolated from *Chamaepsila rosae*) was successful, yet resting spores were not previously found *in vivo* from that host [16].

Most host-pathogen systems within Entomophthoromocyta have only been studied superficially and mostly without emphasis on winter survival, so more examples of strategies and a combination of strategies will surely be found. For example, for a long time, it was suggested that the well-known aphid pathogenic species *Pandora neoaphidis* did not produce resting spores but survived by special structures known as ‘loricoconidia’ [28]. *P*. *neoaphidis* resting spores *in vivo* were recently found [30] thus this fungus may have more than one winter survival strategy.

## 4. Conclusions

We proved that *Entomophthora schizophorae* survives the winter in its overwintering host, adult *Pollenia* species, through a slow disease development and a slow disease transmission among hosts hibernating in unheated attics. This winter survival strategy represents a third way that can be be added to the two known winter survival strategies for fungi from Entomophthoromycota. The documented winter survival here is probably relatively rare, since several conditions must be fulfilled, especially the presence of the susceptible stage of the host in a limited space during winter. We suggest that more attention is given to the winter survival of fungi from Entomophthoromycota since the mechanism for overwintering can be a key factor for initiating epidemic development during the season.
